# Radiological lymph‐node size improves the prognostic value of systemic inflammation index in rectal cancer with pathologically negative nodes

**DOI:** 10.1002/cam4.5761

**Published:** 2023-03-20

**Authors:** Shaoyong Peng, Xiaoxia Liu, Yingjie Li, Huichuan Yu, Yumo Xie, Xiaolin Wang, Jiaming Zhou, Mingxuan Zhu, Yanxin Luo, Meijin Huang

**Affiliations:** ^1^ Department of Colorectal Surgery, The Sixth Affiliated Hospital Sun Yat‐sen University Guangzhou People's Republic of China; ^2^ Department of General Surgery, The Sixth Affiliated Hospital Sun Yat‐sen University Guangzhou People's Republic of China; ^3^ Guangdong Provincial Key Laboratory of Colorectal and Pelvic Floor Disease, The Sixth Affiliated Hospital Sun Yat‐sen University Guangzhou People's Republic of China; ^4^ Guangdong Institute of Gastroenterology, The Sixth Affiliated Hospital Sun Yat‐sen University Guangzhou People's Republic of China

**Keywords:** NLR, node‐negative rectal cancer, radiological lymph node, survival outcome

## Abstract

**Background:**

The relationship between the radiological lymph node (rLN) size and survival outcome in node‐negative rectal cancer is still uncertain. In this study, we aimed to explore the role of enlarged rLN in predicting the survival of node‐negative rectal cancers.

**Methods:**

We retrospectively reviewed the records of 722 node‐negative rectal cancer who underwent curative resection. Factors associated with DFS (disease‐free survival) and CSS (cancer‐specific survival) were assessed with univariate and multivariate analysis. Survival analysis was performed according to presence with or without enlarged rLN. Combining rLN with NLR as a new index‐inflammation immune score (IIS) for predicting survival. Comparing different models to assess the predictive powers.

**Results:**

A total of 119 patients had tumor recurrence and 73 patients died due to cancer. Patients with enlarged rLN (≥5 mm) was significantly associated with better DFS (HR:0.517, 95%CI:0.339–0.787, *p* = 0.002) and CSS (HR:0.43, 95%CI:0.242–0.763, *p* = 0.004). The risk factors of recurrence were rLN, neutrophil‐lymphocyte ratio (NLR), CEA level, and distance from the anal verge. The risk of recurrence increased by 1.88‐ and 2.83‐fold for the high score in IIS compared with the low and intermediate score group (All *p* < 0.001). Similarly, the high score in IIS also increased the risk of cancer‐specific death. In the model comparison, the AIC and LR were improved by including the rLN into the NLR model for DFS and CSS prediction (All *p* < 0.05).

**Conclusions:**

Node‐negative rectal cancer patients with enlarged rLN had a better survival outcome. IIS might be a more comprehensive and complete inflammation immune index for survival prediction.

## 1 INTRODUCTION

Rectal cancer is one of the most common malignancies and a leading cause of cancer‐related mortality.^1^, ^2^ Among them, nearly 15% were Stage I‐II rectal cancer, namely node‐negative rectal cancer. Due to the wider utilization of colonoscopy and the increasing accessibility of screening programs, node‐negative rectal cancer is becoming prevailing in clinical practice. Theoretically, node‐negative rectal cancer can be cured by radical surgery, while, indeed, up to 20% of these patients still suffer from recurrence and have poor outcome.

The inflammation and immune play a crucial role in tumorigenesis and the progression of colorectal cancer.^3^ Many systemic inflammation indexes, such as neutrophil‐lymphocyte ratio (NLR) and platelet‐to‐lymphocyte ratio (PLR), have been suggested as prognostic markers of colorectal cancer in many studies.^4^, ^5^, ^6^ However, their predictive values are still controversial and with high variability,^7^, ^8^ suggesting a dramatic heterogeneity among cancer populations and the predictive accuracy could be improved by combining with other markers.^4^

It has been well established that the number of evaluated lymph nodes (LNs) correlates with better outcomes in node‐negative colorectal cancers.^9^, ^10^ One of the theories to explain this association was an immunological effect.^11^, ^12^ The activation of the antitumor immune response induced the proliferation of lymphocytes and the enlargement of LNs, which makes it easy to harvest the LNs in high numbers. Thus, the enlarged but metastasis‐free LNs could be an indicator of the activation of a local immune response.^13^ Furthermore, the enlarged LN was demonstrated to correlate with a favorable outcome in colon cancer in several studies.^11^, ^14^, ^15^ However, few studies explored the relationships between enlarged LNs and outcomes in node‐negative rectal cancer.

Previous studies have demonstrated the substantial cross‐talk between local immune response and systemic immune inflammation response.^16^ For example, NLR has been found to be correlated with the intratumor neutrophil population and granulocyte myeloid‐derived suppressor cells.^17^, ^18^ Thus, we hypothesized the power of the predictive model would be improved by combining the systemic immune marker with the local immune index (such as LN size), which may present a more comprehensive and complete tumor immune response network.

To date, in most studies investigating the prognostic impact of harvested LN, the size of LN was measured in pathological specimens, which was usually time‐consuming and intensive for a pathologist. Detecting and measuring the LNs in the radiological image was much more feasible and simpler than in specimens. In this study, we aim to explore the prognostic value of radiological LNs in node‐negative rectal cancer. Furthermore, we would like to investigate whether the prognostic model could be improved by combining NLR with rLNs.

## 2 PATIENTS AND METHODS

### 2.1 Patients

**FIGURE 1:**
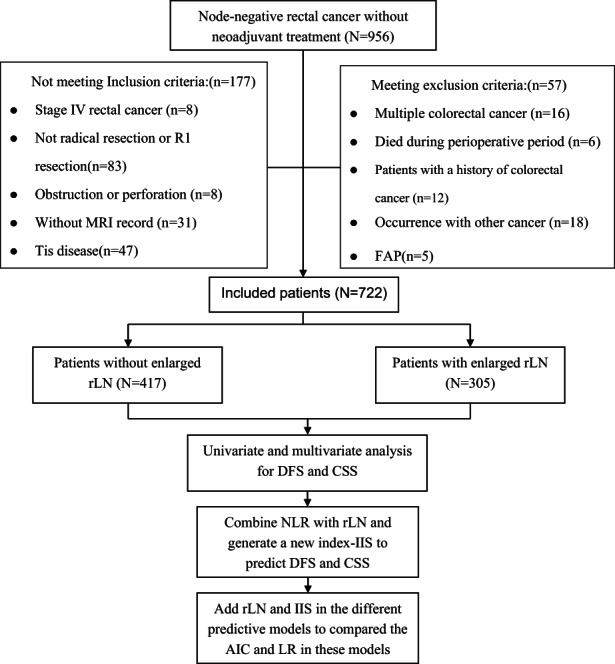
Patient disposition in the analysis of the relationship of rLN and survival outcome. AIC, Akaike information criterion value; CSS, cancer‐specific survival; DFS, disease‐free survival; FAP, familial adenomatous polyposis; IIS, inflammation immune score; LR, likelihood ratio; NLR, neutrophil to lymphocyte rate; rLN, radiological lymph‐node.

The prospectively collected data for consecutive patients in the colorectal cancer database who were node‐negative rectal cancer at the Sixth Affiliated Hospital of Sun Yat‐sen University between March 2010 and July 2017 was reviewed. All patients included in this study had a histologically confirmed adenocarcinoma without lymph node (LN) metastasis and had not received neoadjuvant therapy. Besides, we also excluded patients with obstruction or perforation because the enlarged lymph node might be induced by the severe bacterium infection instead of the tumorous immune response. The other exclusion criteria were as follows (Figure 1): with multi‐origin colorectal cancer or being concurrent with other cancers (including gastric cancer), without MRI record before surgery, with a history of colorectal cancer, or who died during the perioperative period, carcinoma in situ. Patients with total mesorectal excision with R0 resection were included in this study. The surgeries included low anterior resection, abdominoperineal resection, or the Hartman procedure. All the patients have no past medical history, including tuberculosis, autoimmune disease, atopy or HIV infection, and no medication intake or pet ownership history. The MRI, laboratory and follow‐up records were collected. This retrospective study was approved by the Clinical Research Ethics Committee of the Sixth Affiliated Hospital of Sun Yat‐sen University and written informed consent was obtained from all the patients. Patients were followed every 3–6 months with a clinical assessment including a physical examination, serum CEA level and other blood tests, annual imaging tests for abdomen and chest, and then colonoscopy every 2 years. The follow‐up was updated in April 2021 for this study and the failure to respond to our visit for more than 1 year was considered as lost to follow‐up.

### 2.2 MRI protocol

The rectal should be completely free of stool before an MRI scan to further characterize the tumor. Patients would be injected 20 mL of dimeglumine gadopentetate 30–60 s before a rectal MRI scan. With patients in a foot‐first supine position, the coil center was located at the symphysis pubis.

All MRI examinations were performed with a 1.5 T superconducting MRI scanner and an 8 channels phased‐array body surface coil (GE Optima 360, USA). Turbo spin‐echo T2WI sequences and enhanced sequences were acquired in the oblique axial, oblique coronal, and sagittal planes, referred to the rectum (4310 ms repetition time/120 ms echo time, 5 mm section thickness, 280 fields of view, duration 540 s), DWI in the oblique axial, referred to the rectum (4580 ms repetition time/120 ms echo time, 4 mm section thickness), and T1W1 and enhanced sequences in oblique axial (630 ms repetition time/13 ms echo time, 4 mm section thickness).

Images were evaluated by two radiologists using a PACS (picture archive and communication system). The size of lymph nodes was assessed by them individually, blinded to the clinical information. The lymph nodes evaluated in our study are located in the mesorectal, superior rectal, inferior mesenteric, and inferior rectal, the same as the region of total mesorectal excision. An enlarged node was defined as ≥5 mm in a short diameter on MRI.^19^, ^20^ The case would be reviewed and assessed by the third radiologist when there were different opinions on the diagnosis.

### 2.3 Assessment

Before surgery, patients underwent a preoperational assessment, including contrast‐enhanced computer tomography of chest, abdomen and pelvis, contrast‐enhanced MRI of the rectum, colonofiberscopy, and hematologic examination. The cardiac and pulmonary function test was also performed in senile patients to evaluate the risks of the operation. Rectal cancer was defined as the distal edge of the tumor located ≤15 cm from the anal verge. The TNM stage of tumor was staged according to the American Joint Committee on Cancer, seventh edition.

### 2.4 Statistical analysis

The IBM SPSS Statistics 22.0 was used to analyze the data. The Pearson chi‐square was used to compare categorical variables at baseline. The Kaplan–Meier method was used to analyze disease‐free survival and cancer‐specific survival. The log‐rank test was applied to compare the differences. Enter cox proportional hazards model was used in univariate analysis. The forward stepwise Cox proportional hazards model with log‐rank test (multifactorial analysis) was used to evaluate which parameters have an independent effect on survival and the *p* < 0.05 were considered as significant factors. To generate a more comprehensive index of inflammation immune response, we combine the rLN size and NLR into one new variable—inflammation immune score (IIS). According to IIS, the patients were divided into three groups, including high score group (without rLN ≥ 5 mm and NLR ≥ 3), intermediate score group (without rLN ≥ 5 mm or NLR ≥ 3), and low score group (with rLN ≥ 5 mm and NLR < 3). We divided the patients with rLN 5 mm and NLR 3 according to the previous studies.^4^, ^14^ The comparison between different predictive models shown in Table 5 was assessed through Akaike information criterion (AIC) likelihood ratio (LR), as previously described.^7^

## 3 RESULTS

### 3.1 Patient characteristics

A total of 722 consecutive patients meeting the criteria were enrolled in our study with a median follow‐up time of 38 (range:3–127) months. Among them, 119 patients had a tumor recurrence and 73 patients died due to cancer during the follow‐up. Descriptive details in clinical‐pathological parameters were shown in Table 1. There were no significant differences in age, histologic differentiation, positive lymphatic or venous invasion, positive perineural invasion, and the rate of adjuvant chemotherapy between the two groups. Of note, patients with enlarged rLNs were significantly correlated with advanced stage (T3/T4) and more retrieved LNs (All *p* < 0.001). In the systemic inflammatory indexes, no significance in the NLR was observed between the two groups.

**TABLE 1:** Clinical and pathological characteristics of enrolled patients.

Variables	Group without enlarged rLNs (*n* = 417) (%)	Group with enlarged rLNS (*n* = 305) (%)	Overall (*n* = 722) (%)	*p*
Gender				0.040*
Female	182 (43.6%)	110 (36.1%)	292 (40.4%)	
Male	235 (56.4%)	195 (63.9%)	430 (59.6%)	
Age				0.400
<60	195 (46.8%)	133 (43.6%)	328 (45.4%)	
≥60	222 (53.2%)	172 (56.4%)	394 (54.6%)	
Histological type				0.369
Poor, muc	69 (16.5%)	43 (14.1%)	112 (15.5%)	
Well, mod	348 (83.5%)	262 (85.9%)	610 (84.5%)	
Vascular penetration				0.232
Positive	23 (5.5%)	11 (3.6%)	34 (4.7%)	
Negative	394 (94.5%)	294 (96.4%)	688 (95.3%)	
Perineural invasion				0.940
Positive	20 (4.8%)	15 (4.9%)	35 (4.8%)	
Negative	397 (95.2%)	290 (95.1%)	687 (95.2%)	
T stage				<0.001*
pT1/T2	221 (53.0%)	96 (31.5%)	317 (43.9%)	
pT3/T4	196 (47.0%)	209 (68.5%)	405 (56.1%)	
Retrieved lymph node number				<0.001*
<12	66 (15.8%)	13 (4.3%)	79 (10.9%)	
≥12	351 (84.2%)	292 (95.7%)	643 (89.1%)	
NLR				0.072
<3	337 (80.8%)	233 (76.4%)	570 (78.9%)	
≥3	57 (13.7%)	57 (18.7%)	114 (15.8%)	
Missing value	23 (5.5%)	15 (4.9%)	38 (5.3%)	
CEA				0.158
<5	285 (68.4%)	195 (63.9%)	480 (66.5%)	
≥5	102 (24.5%)	89 (29.2%)	191 (26.4%)	
Missing value	30(7.1%)	21(6.9%)	51(7.1%)	
Adjuvant treatment				0.591
Yes	31 (7.4%)	26 (8.5%)	57 (7.9%)	
No	386 (92.6%)	279 (91.5%)	665 (92.1%)	

Statistical significance was defined as P ≤0.05 indicate by * (bold). Abbreviations: CEA, carcinoembryonic antigen; NLR, neutrophil to lymphocyte rat; rLN, radiological lymph‐node; muc, mucinous; mor, moderate.

### 3.2 Univariate and multivariate Cox regression for DFS and CSS

The univariate Cox regression model for DFS and CSS indicated the worse outcomes were significantly correlated with several risk factors, such as T3/T4 stage, perineural invasion, high NLR, elevated CEA level, and tumor close to the anal verge (all *p* < 0.05). The model also showed that patients with enlarged rLNs were associated with a better DFS (*p* = 0.003) and CCS (*p* = 0.005) (Tables 2 and 3). Next, significant variables found in univariate Cox regression were subjected to multivariate Cox regression analysis to determine the independent factors related to DFS and CSS (Tables 2 and 3). In the multivariate model, NLR, CEA level and distance from anal verge were identified as independent variables associated with DFS (all *p* < 0.05). Meanwhile, the enlarged rLN was also an independent prognostic factor and was associated with a better DFS, with an HR of 0.517 (95% confidential interval (CI): 0.339–0.787, *p* = 0.002). The multivariate model for CSS produced a quite similar result but differs in some factors. The enlarged rLN, NLR, and distance from the anal verge were suggested as independent factors in both DFS model and CSS model, whereas perineural invasion was considered as an independent risk factor only in the CSS model (Table 3).

**TABLE 2:** Univariate and multivariate analysis of predictive factors for disease‐free survival.

	Univariate analysis		Multivariate analysis	
Variables	Hazard ratio (95%CI)	*p*‐value	Adjusted hazard ratio (95%CI)	Adjusted *p*‐value
Age		0.555		
≥60/<60	1.115 (0.776–1.602)			
Gender		0.116		
Male/Female	1.357 (0.927–1.984)			
present with rLN ≥5 (mm)		0.003*		0.002*
With/Without	0.551 (0.372–0.818)		0.517 (0.339–0.787)	
T stage		0.027*		0.206
pT3 or T4/pT1 or T2	1.529 (1.050–2.227)		1.308 (0.863–1.982)	
Adjuvant treatment		0.131		
Yes/No	0.501 (0.205–1.228)			
Vascular invasion		0.072		
Positive/Negative	1.866 (0.945–3.682)			
Perineural invasion		<0.001*		0.070
Positive/Negative	2.919 (1.639–5.199)		1.866 (0.951–3.663)	
Histology		0.198		
Poor, muc/Well, mod	1.348 (0.855–2.126)			
White blood cell count, 10^6^/ml		0.565		
≥6.33/<6.33	0.896 (0.616–1.303)			
C reaction protein level		0.329		
≥1.68/<1.68	1.229 (0.812–1.862)			
NLR		0.013*		0.002*
≥3/<3	1.733 (1.122–2.675)		2.016 (1.283–3.169)	
Retrieved lymph node number		0.091		
<12/≥12	1.527 (0.935–2.494)			
CEA		0.003*		0.012*
≥5/<5	1.783 (1.214–2.619)		1.671 (1.121–2.492)	
Distance from anal verge	0.916 (0.871–0.964)	<0.001*	0.913 (0.865–0.962)	<0.001*

Statistical significance was defined as P ≤0.05 indicate by * (bold). Abbreviations: CEA, carcinoembryonic antigen; NLR, neutrophil to lymphocyte rat; rLN, radiological lymph‐node; mor, moderate; muc, mucinous.

**TABLE 3:** Univariate and multivariate analysis of predictive factors for cancer‐specific survival.

	Univariate analysis		Multivariate analysis	
Variables	Hazard ratio (95%CI)	*p*‐value	Adjusted hazard ratio (95%CI)	Adjusted *p*‐value
Age		0.273		
≥60/<60	1.298 (0.814–2.071)			
Gender		0.120		
Male/Female	1.480 (0.903–2.425)			
Present with rLN ≥5 (mm)		0.005*		0.004*
With/Without	0.476 (0.282–0.803)		0.430 (0.242–0.763)	
T stage		0.033*		0.201
pT3 or T4 / pT1 or T2	1.703 (1.045–2.775)		1.433 (0.825–2.488)	
Adjuvant treatment		0.161		
Yes / No	0.366 (0.090–1.494)			
Vascular invasion		0.086		
Positive / Negative	2.082 (0.902–4.804)			
Perineural invasion		<0.001*		0.035*
Positive / Negative	3.967 (2.031–7.750)		2.426 (1.065–5.524)	
Histology		0.421		
Poor, muc /Well, mod	1.270 (0.709–2.275)			
White blood cell count, 10^6^/ml		0.661		
≥6.33/<6.33	0.897 (0.551–1.459)			
C reaction protein level		0.642		
≥1.68/<1.68	1.141 (0.655–1.988)			
NLR		0.012*		0.003*
≥3/<3	2.015 (1.170–3.470)		2.353 (1.334–4.150)	
Retrieved lymph node number		0.190		
<12/≥12	1.513 (0.814–2.812)			
CEA		0.046*		0.145
≥5/<5	1.676 (1.009–2.784)		1.485 (0.872–2.529)	
Distance from anal verge	0.897 (0.840–0.958)	<0.001*	0.892 (0.832–0.957)	<0.001*

Statistical significance was defined as P ≤0.05 indicate by * (bold). Abbreviations: CEA, carcinoembryonic antigen; NLR, neutrophil to lymphocyte rate; rLN, radiological lymph‐node; muc, mucinous; mor, moderate.

### 3.3 Survival analysis for rLN

**FIGURE 2:**
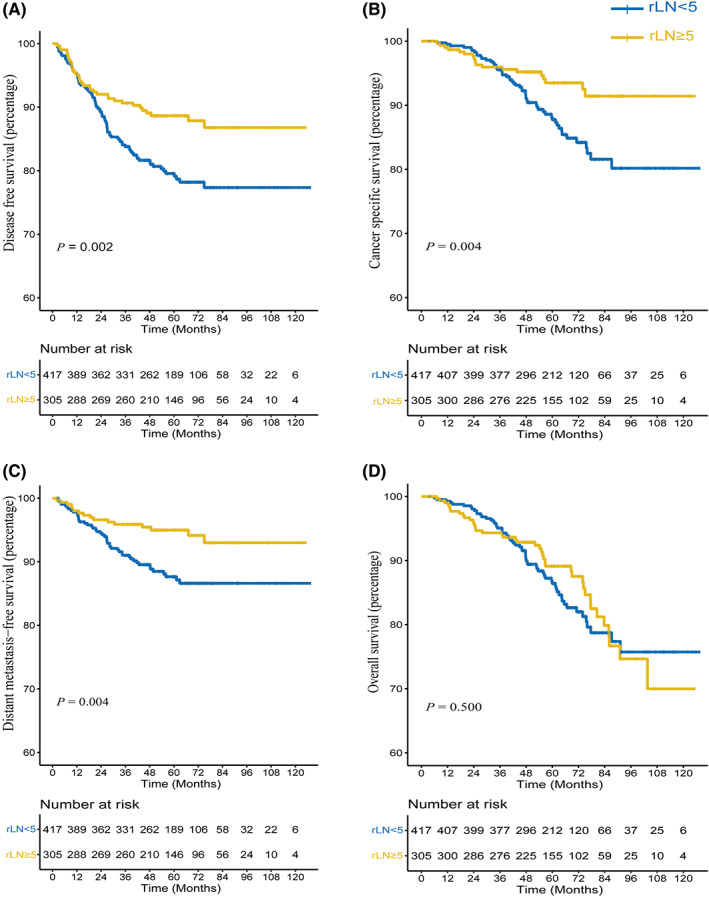
Survival analysis of using the Kaplan–Meier method according to rLN size. The Kaplan–Meier curves for DFS (A), CSS (B), DMFS (C), and OS (D) in patients with or without enlarged rLNs. The log‐rank test was applied and *p*‐values were supplied in each plot.

In this study, the 3‐year disease‐free survival (DFS) rate was 90.7% in the patients with enlarged rLNs, which was significantly better than those without enlarged rLNs (84.1%, *p* = 0.003) (Figure 2A). Moreover, patients with enlarged rLNs had a statistically significant decrease in distant metastasis‐free survival (DMFS) comparing patients without (*p* = 0.004) (Figure 2C). Consistently, the 5‐year cancer‐specific survival rate (CCS) of patients with enlarged rLNs was 93.5%, whereas that of patients without enlarged rLNs was 87.8% (*p* = 0.004) (Figure 2B). However, the overall survival (OS) was similar between the two groups (*p* = 0.501) (Figure 2D).

### 3.4 Generate IIS by combining NLR and rLN and predict DFS and CSS

**FIGURE 3:**
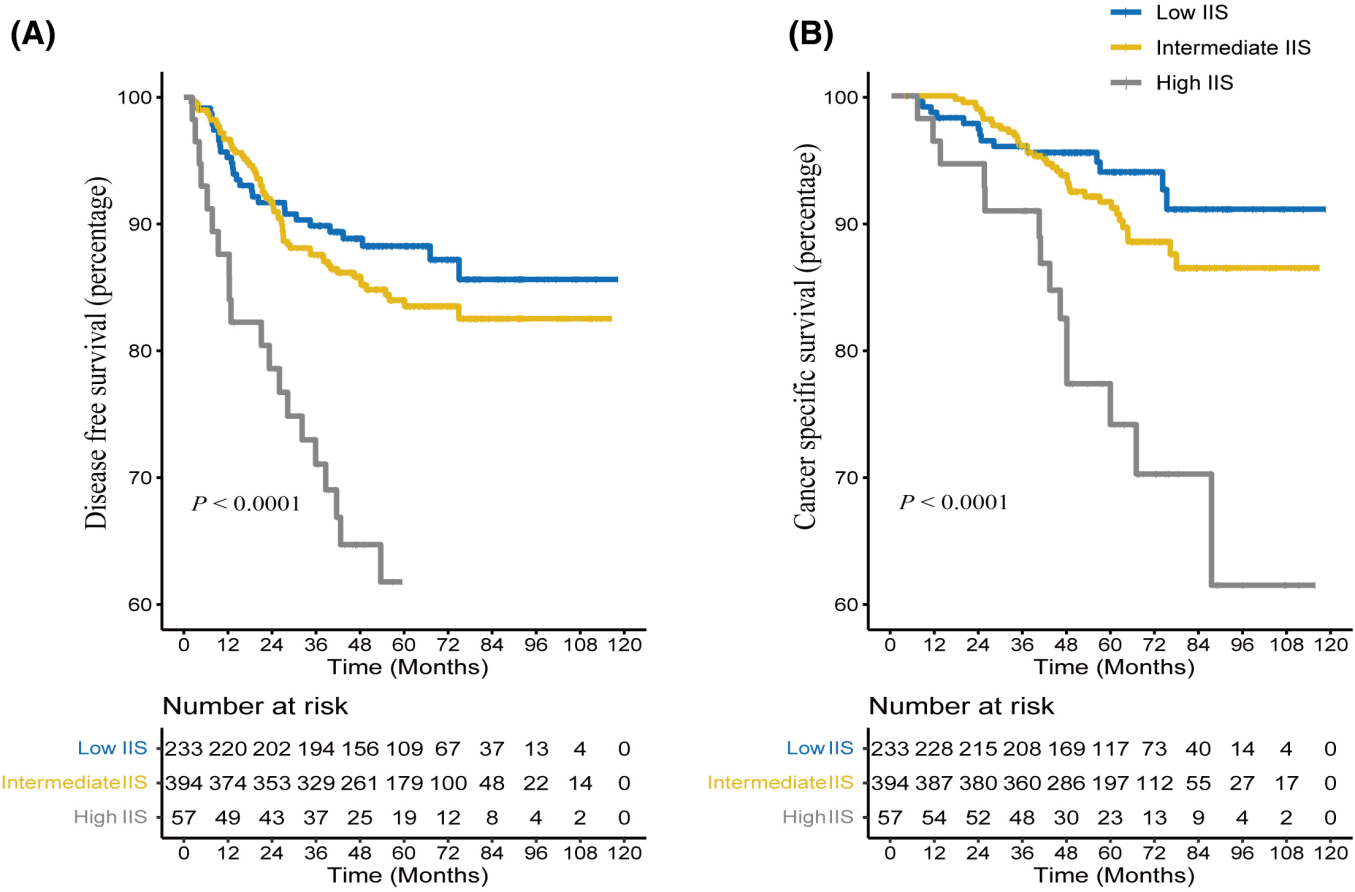
Survival analysis of using the Kaplan–Meier method according to inflammation immune score (IIS). The Kaplan–Meier curves for DFS (A) and CSS (B) in high IIS, intermediate IIS and low IIS.

To generate a more comprehensive and complete immune inflammation marker to predict the survival outcome, the NLR was merged with rLN to form a new maker‐inflammation immune score (IIS). According to the inflammation immune score (IIS) detailed in the method, patients were divided into three groups. Thirty‐eight patients had missing value in IIS because of the missing NLR. In total, 57, 394, and 233 patients were included in the high score group, intermediate score group, and low score group, respectively. The Kaplan–Meier model showed a significant difference in the DFS and CSS curves of the three groups (Both *p* < 0.001, Figure 3). More importantly, a significant difference was also found in the Kaplan–Meier survival analysis for OS, which was not associated with enlarged lymph nodes. (*p* < 0.001, Figure 3). In the Cox regression model for DFS, the hazard ratio for the high score group was 1.888 and 2.831 compared with the intermediate score group and low score group, respectively (95% CI: 1.422–2.505; 1.724–4.65, both *p* < 0.001). Further analysis for CSS showed that patients with high IIS score carried 2.11‐ and 2.97‐fold increased risk of death compared to patients with low IIS and intermediate IIS score, respectively (Table 4).

**TABLE 4:** Univariate analysis of IIS for disease‐free survival and cancer‐specific survival.

	HR	95%CI	*p*
Disease‐free survival			
High IIS compare Low IIS	1.88	1.42–2.50	<0.001*
High IIS compare Intermediate IIS	2.83	1.72–4.6	<0.001*
Cancer‐specific survival			
High IIS compare Low IIS	2.11	1.45–3.06	<0.001*
High IIS compare Intermediate IIS	2.97	1.60–5.49	0.001*

Statistical significance was defined as P ≤0.05 indicate by * (bold). Abbreviation: IIS, inflammation immune score.

### 3.5 rLN improve the predicted value of NLR by merging into IIS

To determine whether the rLN could improve the prognostic value of NLR, we applied likelihood ratio (LR) and AIC to explore how the local immune index (rLN) could improve the systemic inflammation marker (NLR) in predicting the survival outcome. Furthermore, the IIS was also included in the multivariate model to determine if IIS could replace the NLR and rLN as a new marker.

As shown in Table 5 for DFS, model C including the rLN and NLR had a lower AIC value and a higher LR compared to model A or model B which only included NLR or rLN (AIC: 1384.68 vs. 1390.94 and 1389.76; LR: 13.83 vs. 5.57 and 6.75; All *p* < 0.05; Table 5). Further inclusion of the independent prognostic factors mentioned above (CEA, distance from annal verge) in model D and model E, the results also showed that the rLN added in model E significantly improved the predicted value of model D (AIC: 1336.32 vs. 1343.46; LR: 34.21 vs. 25.06; *p* = 0.002, Table 5).

**TABLE 5:** Model fit among six models including or not including rLN or IIS.

Model	Disease‐free survival			Cancer‐specific survival		
	AIC	LR	*p*‐value	AIC	LR	*p*‐value
Model A	1390.94	5.57	0.004	798.72	5.69	0.006^a^
Model B	1389.76	6.75	0.007	798.24	6.17	0.008^b^
Model C	1384.68	13.83		793.26	13.15	
Model D	1343.46	25.06	0.002	759.50	19.21	0.004^c^
Model E	1336.32	34.21		753.41	27.29	
Model F	1334.46	34.06	0.701	751.45	27.25	0.839^d^

*Note*: Model A includes NLR, Model B includes rLN, Model C includes NLR and rLN, Model D includes NLR, CEA, distance from annal verge, Model E includes rLN and all variables in model D, Model F includes CEA, distance from annal verge and inflammation immune score. a, b, *p*‐values of LR test in model A and B compared with model C. c, d, *p*‐values of LR in model D and F compared with model E.

Abbreviations: AIC, Akaike information criterion value; CEA, carcinoembryonic antigen; IIS, inflammation immune score; NLR, neutrophil to lymphocyte; rLN, radiological lymph‐node; LR, likelihood ratio.

Further analysis for CSS, similar results were shown in Table 5. Compared to models A and B, the AIC and LR were significantly improved by adding the rLN into model C (AIC: 793.26 vs. 798.72 and 798.24; LR: 13.15 vs. 5.69 and 6.17; All *p* < 0.05). Even in the presence of other prognostic factors in these models, the rLN also could significantly improve the AIC and LR in the model without it (AIC: 753.41 vs. 759.50; LR: 27.29 vs. 19.21; *p* = 0.004, Table 5). Thus, the rLN could significantly improve the predictive value of NLR regardless of the presence of other prognostic factors or not.

Models E and F were built to explore the predictive value of ISS further. The predicted value of model E and model F was nearly the same when rLN and NLR were replaced with IIS in model F (AIC: 1336.32 vs. 1334.46; LR: 34.21 vs. 34.06; *p* = 0.701, Table 5). Similar results were also found in the analysis for CSS (Table 5). All these results showed that the rLN could improve the predicted value of NLR, and IIS may represent the rLN and NLR as a comprehensive inflammation immune index in predicting survival outcomes.

## 4 DISCUSSION

The enlarged rLN was commonly neglected by surgeons after surgery, especially in node‐negative patients. In this study, we found that enlarged rLN was significantly associated with a better DFS and CSS. In addition, the rLN as a local immune index could improve the predicted value of NLR. The rLN and NLR could be combined as a new index‐inflammation immune score (IIS) in the models for predicting PFS and CSS and IIS could predict OS which could not be predicted by rLN and NLR. All these results showed the important role of rLN in node‐negative rectal cancer.

One study of our center had reported that the enlarged rLN was a risk factor in colon cancer. Patients with enlarged rLN had poor DFS and OS because of micrometastasis of these LNs.^21^ Bruno and his colleagues demonstrated that node‐negative colon cancer with more than seven enlarged LN (>5 mm) had a significantly better outcome comparing those without.^22^ These contrasting results showed that the prognostic value of LN in colorectal cancer remained unclear and additional studies with a larger sample size were necessary. To our knowledge, this is the largest study to evaluate the prognostic value of rLN, and we demonstrated that enlarged rLN was associated with a better survival outcome in node‐negative rectal cancer.

Usually, tumor recurrences were divided into local recurrence and distant metastasis. Among them, the local recurrence was attributed to the microscopic residual tumor, whereas distant metastases resulted from the cancer cells which traveled through the blood and lymphatic systems. Thus, the presence of LN metastasis was considered as the precursor of distant metastases in colorectal cancer.^23^, ^24^ Naxerova et al. observed that one‐third of colorectal cancers shared a common sub‐clonal origin between distant metastases and LN metastases.^25^ In this study, we illustrated that patients with enlarged rLNs had better distant metastasis‐free survival. The results may indicate that proliferation and activation of lymphocytes in the enlarged LN could prevent metastasis by improving the cancer cell elimination which was transported from lymphatic systems.^26^ Of course, this conclusion remains to be further investigated in cell biology.

As well as metastasis prevention, the LN is an organ to generate tumor‐infiltrating lymphocytes when the tumor antigens reach it to stimulate T cells.^27^, ^28^ The activation and proliferation of lymphocytes may result in the reactive enlargement of LN, reflect an active antitumor response, and thus indirectly impact survival.^29^ More direct evidence was found in Bruno's study in which the positive association between tumor‐infiltrating lymphocytes density and the LN size was shown.^30^ Though there was a difference between rLN and pathological LN, the enlarged rLN predicted a better survival outcome might also be attributed to the active immune response. A more detailed association among immune response, LN size and survival outcomes needs further investigation.

Recently, NLR was combined with many other immune inflammation indexes and created a new marker to predict the survival outcome.^31^ Hirahara and his colleagues combined the NLR with PLR and created the NLR‐PLR score as a new scoring system. In their study, gastric cancer patients with higher NLR‐PLR were significantly associated with poor overall survival.^32^ Also, in a big cohort that included 1383 colorectal cancer with radical surgery, the immune‐inflammation index was generated by combining platelet count with NLR and predicted a poor DFS and OS.^33^ In our study, rLN was combined with NLR, created IIS as a new marker and found IIS was significantly associated with DFS, CSS, and OS.

Moreover, IIS was a more effective predictor of survival outcomes compared to NLR or rLN based on the LR and AIC values. As shown in Table 5, the IIS could represent NLR and rLN to predict the survival outcomes in node‐negative rectal cancer. The potential explanation for a better prognostic value might be that IIS was a more comprehensive index reflecting the inflammatory immune response. This combination improved the predictive value of NLR, which implied that NLR may also be only one part of the systemic immune response and why not powerful enough to predict the survival outcome in some studies.^34^

There are a few limitations to this study. First, it was a single‐center, retrospective study and a large‐scale prospective validation study to investigate the conclusion was required. Second, rLN size might be easily affected by different types of MRI machines. Therefore, the cut‐off point of rLN size might vary in different centers, impacting its wild application. Third, the MRI examinations may miss some enlarged lymph nodes resulting in an inaccurate rLN stage. A more comprehensive evaluation of the lymph nodes may be performed in the next study to improve the accuracy of rLN stage. Last, though the combination of NLR with other immune indexes was reported in several studies, few combined NLR with Rln. Thus the prognostic value of IIS needs to be further validated in more studies.

## 5 CONCLUSION

On the whole, enlarged but pathologically negative rLN in rectal cancer might represent the activation of patients' antitumor immune. Thus, node‐negative rectal cancer patients with enlarged rLN have a better survival outcome than those without. Furthermore, by combining rLN with NLR as IIS, this new index could predict the survival outcome more effectively. These results need further validation in other studies and evaluate the need for adjuvant therapy in patients with high IIS.

## AUTHOR CONTRIBUTIONS

**Peng Shaoyong:** Conceptualization (lead); data curation (lead); investigation (equal); methodology (equal). **Xiaoxia Liu:** Investigation (equal); methodology (equal). **Yinjie Li:** Conceptualization (supporting); data curation (supporting). **Huichuan Yu:** Writing – original draft (supporting); writing – review and editing (supporting). **Yumo Xie:** Writing – original draft (supporting); writing – review and editing (supporting). **Xiaolin Wang:** Validation (lead); visualization (lead). **Jiaming Zhou:** Validation (supporting); visualization (supporting). **Mingxuan Zhu:** Resources (lead); software (lead). **Yanxin Luo:** Funding acquisition (lead); supervision (lead). **Meijin Huang:** Writing – original draft (lead); writing – review and editing (lead).

## FUNDING INFORMTION

This study was supported by the National Natural Science Foundation of China (No.82173067, YL; No.81972245, YL; No.81902877, HY), the Natural Science Foundation of Guangdong Province (No.2022A1515012656, HY; No.2021A1515010134, MH; No. 2020A1515010036, XL), the Sun Yat‐sen University Clinical Research 5010 Program (No. 2018026, YL), the “Five Five” Talent Team Construction Project of the Sixth Affiliated Hospital of Sun Yat‐sen University (No.P20150227202010251, YL), the Excellent Talent Training Project of the Sixth Affiliated Hospital of Sun Yat‐sen University (No. R2021217202512965, YL), the Sixth Affiliated Hospital of Sun Yat‐sen University Clinical Research‐’1010’ Program (MH), the Program of Introducing Talents of Discipline to Universities, and National Key Clinical Discipline (2012).

## CONFLICT OF INTEREST STATEMENT

The authors declare no conflict of interest.

## ETHICS STATEMENT

Before conducting this study, we obtained a review board approval from the Sixth Affiliated hospital of Sun Yat‐sen University.

## INFORMED CONSENT

The written informed consent was obtained from all the patients in this study.

## Data Availability

The data from the SYSU cohort that support the findings of this study are available on request from the corresponding author.
